# Hierarchical Involvement of Myeloid-Derived Suppressor Cells and Monocytes Expressing Latency-Associated Peptide in Plasma Cell Dyscrasias

**DOI:** 10.4274/tjh.2018.0022

**Published:** 2018-05-25

**Authors:** Tamar Tadmor, Ilana Levy, Zahava Vadasz

**Affiliations:** 1 Bnai-Zion Medical Center, Clinic of Hematology, Haifa, Israel; 2 The Ruth and Bruce Rappaport Faculty of Medicine, Clinic of Hematology, Haifa, Israel; 3 Bnai-Zion Medical Center, Clinic of Internal Medicine B, Haifa, Israel; 4 Bnai-Zion Medical Center, Clinic of Allergy and Clinical Immunology, Haifa, Israel

**Keywords:** Multiple myeloma, Monoclonal gammopathy of unknown significance, Myeloid-derived suppressor cells, Latency-associated peptide

## Abstract

**Objective:**

Plasma cell dyscrasias (PCDs) are disorders of plasma cells having in common the production of a monoclonal M-protein. They include a spectrum of conditions that may represent a natural progression of the same disease from monoclonal gammopathy of unknown significance to asymptomatic and symptomatic multiple myeloma, plasma cell leukemia, and Waldenström’s macroglobulinemia. In PCDs, the immune system is actively suppressed through the secretion of suppressive factors and the recruitment of immune suppressive subpopulations. In this study, we examined the expression of two subpopulations of cells with immunosuppressive activity, monocytic myeloid-derived suppressor cells (MDSCs) and monocytes expressing latency-associated peptide (LAP), in patients with different PCDs and in healthy volunteers.

**Materials and Methods:**

A total of 27 consecutive patients with PCDs were included in this study. Nineteen healthy volunteers served as controls.

**Results:**

We observed a hierarchical correlation between disease activity and the presence of monocytes with immunosuppressive activity.

**Conclusion:**

These results suggest that MDSCs and monocytes expressing LAP have diverging roles in PCDs and may perhaps serve as biomarkers of tumor activity and bulk.

## Abstract (tr)

**Amaç:**

Plazma hücreli diskrazi (PHD), monoklonal M-proteinin üretimine sahip olan plazma hücrelerinin bozukluklarıdır. Aynı hastalığın, önemi bilinmeyen monoklonal gammopatiden, asemptomatik ve semptomatik multipl myeloma, plazma hücreli lösemi ve Waldenström makroglobulinemiye doğru doğal ilerlemesini temsil edebilen bir dizi spektrum içerir. PHD’lerde, baskılayıcı faktörlerin salgılanması ve bağışıklık baskılayıcı alt popülasyonların katılımı ile bağışıklık sistemi aktif olarak baskılanır. Bu çalışmada, PHD’lerin ve sağlıklı gönüllülerin, immün baskılayıcı aktiviteye sahip iki alt popülasyonundaki; monositik myeloid kökenli baskılayıcı hücreler (MKBH) ve latent asosiye peptit (LAP) eksprese eden monositlerin, ekspresyonunu incelenmiştir.

**Gereç ve Yöntemler:**

Bu çalışmaya PHD’li toplam 27 hasta dahil edildi. On dokuz sağlıklı gönüllü, kontrol olarak kullanılmıştır.

**Bulgular:**

Hastalık aktivitesi ile immünosüpresif aktivitesi olan monositler arasında hiyerarşik bir ilişki gözlenmiştir.

**Sonuç:**

Bu sonuçlar LAP anlatımı gösteren MKBH’lerin ve monositlerin, PHD’lerde farklı rollere sahip olduğunu ve tümör aktivitesi ve kitle biyobelirteçleri olarak kullanılabileceğini düşündürmektedir.

## Introduction

Myeloid-derived suppressor cells (MDSCs) are a heterogeneous population of immature cells of granulocytic or monocytic origin, which accumulate in a number of disorders including solid tumors and hematological malignancies in particular [1,2]. MDSCs inhibit T-cell proliferation and cytokine secretion, favoring the recruitment of regulatory T cells (Tregs), and are part of the immune regulatory subpopulations of cells responsible for inhibition of the immune response, thereby facilitating tumor escape [1,2]. 

Latency-associated peptide (LAP) is the N-terminal propeptide of the transforming growth factor beta (TGF-β) precursor, which binds noncovalently to TGF-β, forming a latent TGF-β complex. When released into the extracellular milieu, LAP forms small latent complexes with TGF-β1 [3,4,5]. TGF-β-LAP complexes are present on the surface of various immune cells and have been shown to play a role in immune regulation, promoting the conversion of naive to activated Tregs, which induce Treg-associated immunosuppression [3,4,5].

Bolzoni et al. [6] studied the function of CD14/CD16+ monocyte subpopulations sorted from the bone marrow of patients with monoclonal gammopathies at different stages of disease. In this report, monocytes isolated from patients with multiple myeloma (MM) showed activity that contributed to enhanced osteoclast activation.

MM is the second most common hematological malignancy in the United States and is invariably preceded by monoclonal gammopathy of unknown significance (MGUS). Myeloma cells are critically dependent on the tumor microenvironment for their survival, progression, and proliferation, and a number of recent studies have concentrated on targeted therapy of tumor niche pathways [7,8,9].

MM is also associated with immune dysfunction, and several reports have demonstrated increased numbers of MDSCs in the bone marrow microenvironment, which contributes to immunosuppression and tumor invasion [10,11,12,13,14,15,16]. 

Recently, we studied two immune subpopulations, monocytic MDSCs and LAP-expressing monocytes, in the peripheral blood of patients with different plasma cell dyscrasias (PCDs) and in healthy volunteers and compared their frequencies.

## Materials and Methods

A total of 27 consecutive patients with PCDs, classified according to the International Myeloma Working Group as published in 2009 and updated in 2014-2015 [14,15] and seen in the Hematology Unit of the Bnai Zion Medical Center in Haifa, Israel, between 2013 and 2015 were included in this study. For patients with plasma cell leukemia, diagnosis was based on the percentage (≥20%) and absolute number (≥2x10^9^/L) of plasma cells in the peripheral blood, while Waldenström’s macroglobulinemia (WM) was defined on the basis of the presence of immunoglobulin M monoclonal gammopathy and ≥10% bone marrow lymphoplasmacytic infiltration [17,18,19,20].

The cohort included 8 patients with MGUS, 14 with symptomatic MM, 2 with plasma cell leukemia, and 3 with WM. Nineteen healthy volunteers served as controls. 

All samples were taken from treatment-naive patients, before starting any therapy.

Written informed consent was obtained from all patients and the study was approved by the hospital’s ethics committee.

### Materials

Mononuclear cells were enriched from whole blood using the Ficoll-Hypaque gradient (Lymphoprep, Oslo, Norway). Fluorescence-activated cell sorting analysis was performed on these mononuclear cells using the following antibodies: anti-CD45 PC-5 (PE-Cy5), anti-CD14 PE (phycoerythrin), and anti-HLA-DR FITC (fluorescein) (BD Biosciences, San Jose, CA, USA). 

For staining, 0.5-1x10^6^ mononuclear cells were stained and incubated at room temperature for 30 min in the dark with the above antibodies according to the manufacturer’s instructions in 100 µL of PBS followed by red blood cell lysis (VersaLyse, Beckman Coulter, Inc., Marseille, France). In addition, MDSCs were characterized using antibodies to CD124 [interleukin (IL)-4Ra], which is the common receptor for interleukin-4 (IL-4). CD14+/HLA-DR^neg/low^ cells were also gated for expression of LAP using anti-LAP (clone 27232), obtained from R&D Systems (Minneapolis, MN, USA).

Data were acquired with a Beckman Coulter Cytomics FC 500 flow cytometer and analyzed with CXP Software, version 2.2. (Beckman Coulter, Brea, CA, USA).

### Statistical Analysis

All values were expressed as mean ± standard error of the mean. For flow-cytometry data, values between groups of data were tested for statistical significance.

The chi-square test was performed to determine whether data were normally distributed and a two-tailed t-test was then applied to the results. Significant p-values were those less than 0.05.

## Results

The patient cohort included 11 males (41%) and 16 females (59%); median age at diagnosis was 61 years (range: 45-86). All patients were diagnosed and followed at the same medical center. Patients’ characteristics are presented in Table 1.

### Monocytic MDSC Expression

The mean number of circulating monocytic MDSCs in the peripheral blood was defined by coexpression of positive CD14+ and dim expression of HLA-DR. The average expression was 5.9% (3.7%-8.1%) for the MGUS cohort, 12.5% (6.7%-27.2%) for MM patients, 18.4% (14.6%-22%) in plasma cell leukemia cases, 17.8% (16.5%-19%) in WM cases, and 5.5% (2.4%-7.9%) in healthy controls.

**Figure 1:**
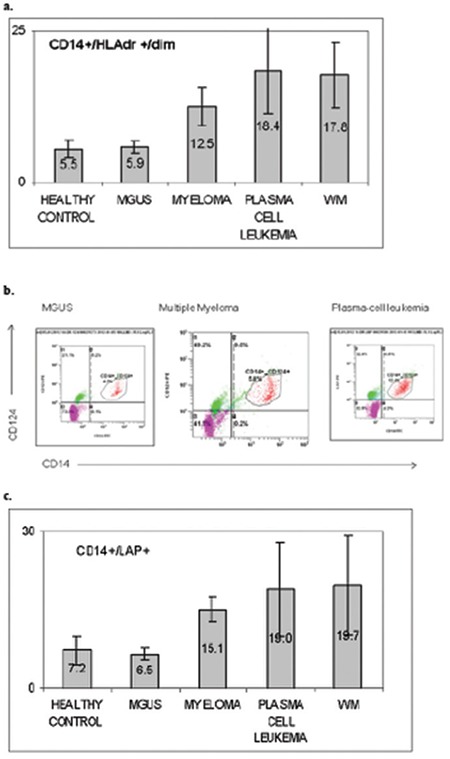
Flow-cytometry analysis of peripheral blood from patients with different plasma cell dyscrasias in comparison to healthy controls. a) Coexpression of CD14+/HLA-DR+dim. b) Coexpression of CD14+/CD124+, both representing the average of myeloid-derived suppressor cell (MDSC) percentage identified in the peripheral blood of each cohort. c) An example of fluorescence activated cell scanning analysis presenting peripheral blood infiltrated by MDSCs in monoclonal gammopathy of unknown significance, multiple myeloma, and plasma cell leukemia patients. MM: Multiple myeloma, MGUS: monoclonal gammopathy of unknown significance, MDSC: Myeloid-derived suppressor cell, LAP: latency-associated peptide, WM: Waldenström’s macroglobulinemia.

No significant difference was observed between MGUS patients and healthy volunteers (p=0.39), but the comparison with cases of PCD was significant (p=0.002) (Figure 1a). Next, we analyzed the monocyte subpopulation coexpressing CD124+, another marker of MDSCs. Results obtained using mean numbers for healthy controls and patients with MGUS, MM, plasma cell leukemia, and WM were 8.1% (6.1%-11%), 4.4% (1.6%-7.1%), 15.7% (2.5%-17.5%), 18.4% (14.5%-22.3%), and 19.7% (18.5%-20.9%), respectively (Figure 1b).

Results were statistically significant for all PCDs when compared to healthy controls (p=0.03).

### LAP Expression

**Figure 2:**
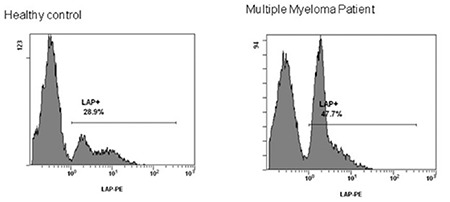
Flow-cytometry analysis of peripheral blood from patients with different plasma cell dyscrasias in comparison to healthy controls for the expression of latency-associated peptide (LAP) on monocytes. a) Coexpression of CD14+/ LAP+. Results represent the average percentage identified in the blood of each cohort. b) An example of fluorescence activated cell scanning analysis presenting peripheral blood infiltrated by monocytes/LAP+ cells in a healthy control and a multiple myeloma patient. LAP: Latency-associated peptide.

The mean number of circulating monocyte/LAP+ cells in the peripheral blood was defined by coexpression of positive CD14+ and LAP. The average expression was 6.5% (3.7%-9.1%) for the MGUS cohort, 15.1% (12.1%-44%) for MM patients, 19% (13.5%-23.2%) in plasma cell leukemia cases, 19.7% (16.9%-23%) in WM cases, and 7.2% (5.9%-9.5%) in healthy controls. No significant difference was observed between MGUS patients and healthy volunteers (p=0.8), but results were significant for other PCDs (p=0.018) (Figures 2a and 2b).

## Discussion

Substantial advances in understanding the biology of PCD progression have been achieved through the study of the bone marrow microenvironment [8]. The bone marrow niche appears to play an important role in the differentiation, proliferation, migration, and survival of plasma cells. It is composed of a heterogeneous cellular compartment that includes stromal cells, osteoblasts, osteoclasts, endothelial cells, and immune cells [13]. Intercellular interaction appears to induce immune dysfunction, which is also an important feature of MGUS and MM and may promote progression from a premalignant state to malignancy [8,10,21,22,23].

Monocytes, macrophages, and mesenchymal stromal cells play a role in MM pathogenesis, where they support the survival and proliferation of neoplastic myeloma cells [25,26,27]. 

MDSCs are a heterogeneous population of immature myeloid cells at different stages of maturation; they play a role in cancer tolerance and function as an immunosuppressive cell subpopulation [2].

Several studies have analyzed the frequency and function of MDSCs in MM, indicating that they promote both myeloma growth and osteoclast activity and are involved in cross-talk with Treg cells, resulting in their expansion in the bone marrow microenvironment [28,29,30,31].

We hypothesize that the enhanced activity of a monocyte subpopulation with immunosuppressive activity may play a role in patients with PCDs. We were able to demonstrate that, in parallel to disease progression from MGUS to MM and plasma cell leukemia, the number of monocytic MDSCs appears to increase and they may express more IL-4R, which is critical for suppression of MDSC function through the L4Ra-STAT6 pathway and thereby indicative of greater immune-related activity [32].

The preliminary results that we report here are in keeping with those of a recent study that also demonstrated increased activity of CD14/CD16+ monocytes in different monoclonal gammopathies in a hierarchical pattern. Indeed, these CD14/C16+ monocytes isolated from MM patients appear to contribute to bone disease and osteoclastogenesis via IL-21 overexpression [6]. 

Recently, a novel regulatory cell subset population has also been described: Tregs and immature dendritic cells that express human LAP(LAP+) [3,4,5,33,34,35]. To date, LAP+ expression on monocytes or monocytic MDSCs has not yet been studied extensively, but based on our lab’s preliminary results, showing high expression of LAP on the surface of CD14+ mononuclear cells isolated from patients with ankylosing spondylitis [35], we decided to examine this phenomenon in patients with PCDs. Here we indeed show that monocytes isolated from these patients have higher positive expression of LAP and that the frequency of its expression was correlated with disease progression.

Our results may have additional significance for biomarkers of disease activity and we are currently initiating a study analyzing these two subpopulations after therapy in symptomatic patients with PCDs.

In addition, it has been reported that when effective therapy for PCD is given, as with immunoregulatory lenalidomide [36,37,38,39] and more recently treatment with daratumumab [40], immunosuppressive MDSCs, Tregs, and Bregs are reduced while the expression of CD4+ T-helper cells and CD8+ cytotoxic T cells is increased, supporting a numerical correlation between their frequency and disease activity.

Our study obviously has several limitations, including the limited size of the cohort, the fact that these immunosuppressive populations were isolated from peripheral blood and not bone marrow, and the lack of functional assays.

## Conclusion

In conclusion, we observed a hierarchical correlation between the subtypes of PCD categories and the recruitment of two subpopulations of monocytes, monocytic MDSCs and monocytes expressing LAP, with immunosuppressive activity. These results suggest that MDSCs and LAP play diverging roles in PCDs and may have potential roles as markers of tumor activity. Our results require further validation and we are now performing a subsequent study to validate them and analyze the effect of therapy on these two subpopulations.

## Figures and Tables

**Table 1:**
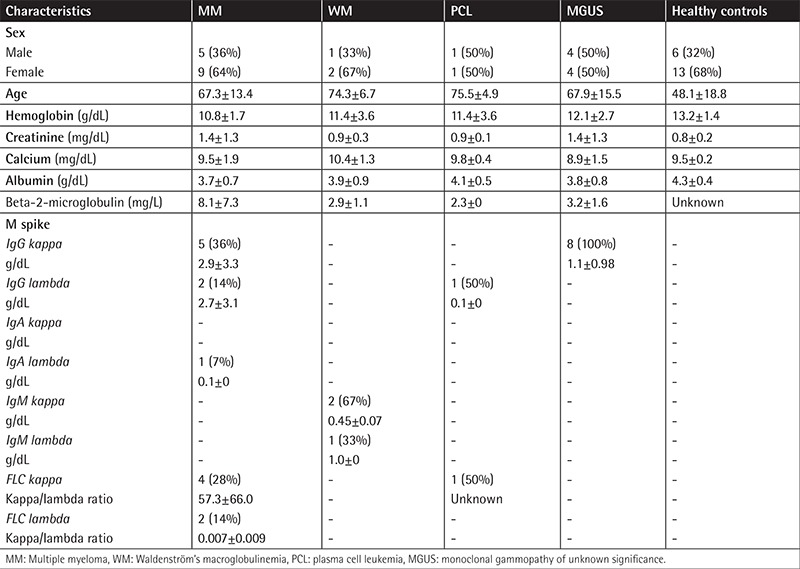
Patients’ demographic, clinical, and laboratory characteristics.
